# Free-breathing cardiovascular magnetic resonance flow quantification can be an alternative to standard breath-holding approach

**DOI:** 10.1038/s41598-025-06126-2

**Published:** 2025-06-20

**Authors:** Sinsia A. Gao, Odd Bech-Hanssen, Christina Polte, Kerstin M. Lagerstrand, Christian L. Polte

**Affiliations:** 1 Department of Clinical Physiology, Sahlgrenska University Hospital, Gothenburg, Sweden; 2 Department of Cardiology, Sahlgrenska University Hospital, Gothenburg, Sweden; 3 Department of Diagnostic Radiation Physics, Sahlgrenska University Hospital, Gothenburg, Sweden; 4 Pediatric Heart Centre Gothenburg, Queen Silvia Childrens Hospital, Sahlgrenska University Hospital, Gothenburg, Sweden; 5 Institute of Medicine, The Sahlgrenska Academy at the University of Gothenburg, Gothenburg, Sweden

**Keywords:** Phase contrast flow quantification, Valvular heart disease, Cardiovascular magnetic resonance, Breath-hold, Free-breathing, Cardiology, Medical research

## Abstract

Cardiovascular magnetic resonance (CMR) evaluation of valvular heart disease is an important diagnostic tool when echocardiography is inconclusive. Phase contrast flow quantification is usually performed during breath hold (BH), which can be challenging in patients suffering from dyspnea and heart failure. The purpose of the present study is to compare a free-breathing (FB) with the conventional BH approach for flow quantification in the aortic, pulmonary and tricuspid valves in 20 healthy subjects (HS) and 25 patients with tricuspid regurgitation (TR). Aortic (AoFF) and pulmonary forward flow volume (PuFF), and tricuspid inflow volume (TrIF) were evaluated. Mean, standard deviation (SD) and limits of agreement (LoA) were calculated. There were good agreements between phase contrast flow volumes obtained by FB and BH approach. Mean difference ± SD / LoA for AoFF during BH versus FB were 1 ± 6 / -10 to 13 ml. The corresponding for PuFF were 1 ± 6 / -11 to 13 ml, and for TrIF − 3 ± 6 / -15 to 9 ml, respectively. Thus, free-breathing CMR flow acquisition can be an important alternative in the assessment of stroke volume, valvular regurgitant volume and be useful in all patients with difficulties to hold their breath.

## Introduction

Valvular heart disease (VHD) is a growing cause of cardiovascular morbidity and mortality worldwide^1^. In the last decades, improved patient survival and transcutaneous procedures accessible to patients previously ineligible for surgery have further contributed to an increased interest in this population. Severity grading is a crucial step in the management of patients with VHD, particularly regarding decision to intervention and follow-up^2^. In cases of an inconclusive echocardiographic evaluation, which is the primary diagnostic modality for VHD, cardiovascular magnetic resonance (CMR) imaging is used as an important complementary secondary diagnostic tool^3^. However, CMR protocols with an oftentimes long scan time and multiple breath-holds can be challenging for patients with heart failure symptoms. Their incapability to hold breath for longer time periods may jeopardize image quality. A free-breathing (FB) approach is hence advantageous in elderly frail patients and patients with dyspnea. Furthermore, the flow measurements in tricuspid and pulmonary valves are prone to respiratory variations compared with the left heart valves. The conventional CMR approach with the acquisition limited to expiratory breath-holding (BH) is less physiologic and could therefore have impact on the quantification of tricuspid regurgitation (TR). However, systematic evaluation in the context of VHD with comparisons between the flow quantifications using FB and BH approaches in multiple valve positions is scarce^4^and no previous studies have performed inter-valvular comparisons with focus on the tricuspid valve.

Thus, the present study aims to evaluate the interchangeability of a FB approach with the conventional BH approach for CMR flow volume quantifications in multiple valve positions in healthy subjects and patients with TR.

## Methods

### Study population

This study includes a group of healthy subjects and a subset of patients with TR from a prospective multi-modality diagnostic study of TR. The healthy subjects (*n* = 20) were recruited among the students and the staff members of the Department of Clinical Physiology and Cardiology at the Sahlgrenska University Hospital. The purpose of enrolling the healthy subjects was to perform an internal precision control of BH and FB flow volume measurements in three valvular positions used for TR quantification by CMR, although the comparison of the tricuspid inflow volume quantification between the two breathing approaches was more important, since it is a less established method than the forward flow measurements in the semilunar valves. Patients with moderate or severe TR (*n* = 25) according to echocardiographic evaluation were recruited from outpatients or patients evaluated prior to intervention at our department. Echocardiography was used for screening and was performed within 2 hours to CMR in all participants. The exclusion criteria for TR patients were the presence of more than moderate regurgitation in any other valves or an intra-cardiac shunt, and irregular heart rhythm. All healthy subjects had no evidence of VHD, intra-cardiac shunt or other cardiovascular diseases.

The study was conducted according to the Declaration of Helsinki. The Regional Ethics Board at the Sahlgrenska University Hospital in Gothenburg gave ethical approval for the study protocol, and written informed consent was obtained from all participants.

### CMR image acquisition

CMR imaging was performed using a 1.5 Tesla scanner (Ingenia, Philips Healthcare, Best, The Netherlands) with an anterior torso and posterior bed coil (dStream). After standardized patient-specific planning, a serie of cine-images was acquired, first in the short-axis (SA) view covering the whole heart without gap from the atrioventricular ring to the apex, followed by cine-images in the common long-axis projections including the left ventricle (LV) and right ventricle (RV) outflow tract view and RV 2-chamber view. All cine-images were acquired using balanced steady-state free precession sequences (repetition time 2.7–3.4 ms, echo time 1.3–1.7 ms, flip angle 60°, echo train length 16) with retrospective ECG gating (30 phases per cardiac cycle) and parallel imaging (acceleration factor 2) during gentle expiratory breath-hold. Typical field of view and in-plane spatial resolution were 290 × 290 mm 1.7 × 1.7 mm, with a slice thickness of 8 mm.

**Fig. 1:**
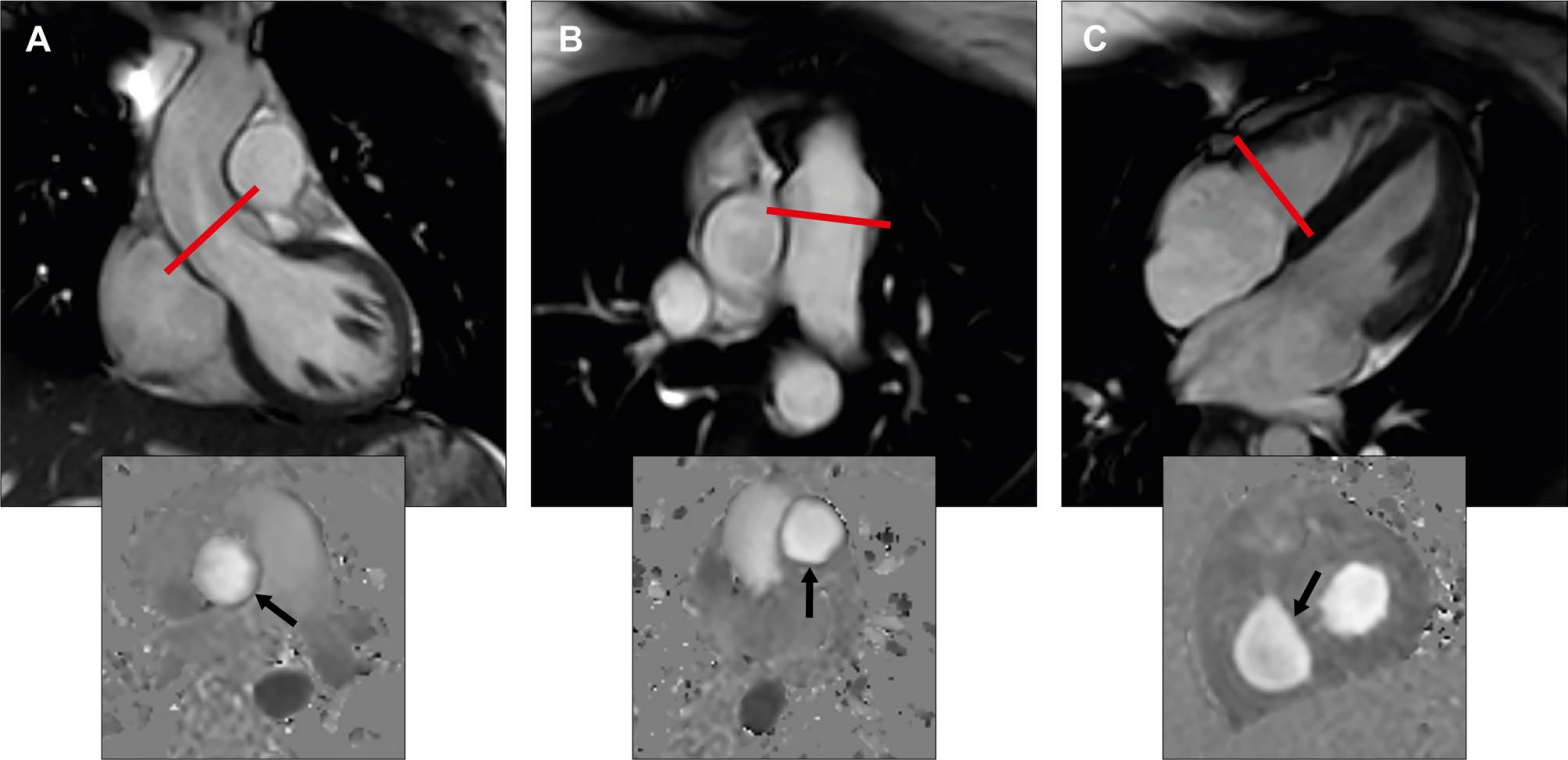
Image acquisition and analysis for CMR flow volume quantification. (**A**,** B**) Left and right ventricular outflow tract view in end-diastole. The red line illustrates the slice position for through plane phase contrast velocity imaging. Quantification of aorta and pulmonary flow is depicted in the corresponding phase contrast image below (arrow). (**C**) Four -chamber view in early diastole with an open tricuspid valve. The red line illustrates the slice position for through plane phase contrast velocity imaging. The corresponding phase contrast image for tricuspid inflow quantification in diastole is shown below (arrow).

Flow volume quantification of the aortic (AoFF) and the pulmonary forward flow (PuFF) as well as the tricuspid inflow (TrIF) was performed using through-plane phase contrast (PC) velocity encoded sequences (repetition time 4.8 ms, echo time 2.9 ms, flip angle 12°, 40 phases per cardiac cycle, echo train length 3, field of view 320 × 260 mm, voxel size 2.5 × 2.5 mm, slice thickness 8 mm) with retrospective ECG gating during gentle expiratory breath-hold for approximately 12–16 s according to the standard BH protocol. The PC images were acquired perpendicularly aligned to the direction of the blood flow in two imaging planes in the aortic root at the level of the sinotubular junction in end-diastole and the main pulmonary artery just above the pulmonary valve (Fig. 1). AoFF was evaluated as an internal precision control to PuFF. In a previous study we have demonstrated high feasibility and reproducibility in the mitral position and in the present study the similar approach was applied to the flow quantification in the tricuspid position^5,6^. PC images were acquired perpendicularly aligned to the direction of the blood flow of tricuspid inflow in three orthogonal planes (4 chamber, 2 chamber and inflow-outflow views of the RV) approximately 1 cm below the tricuspid annulus in early diastole (Fig. 1)^7^. Flow volume quantification was also performed during FB for in average approximately 3 min during gentle free respiration (averaged 24 acquisitions phase-matched to ECG). PC images were acquired twice during BH at all valve positions with an optimized VENC (results are presented as the mean of the two independent measurements) and once during FB in the same position of each valve as during the BH approach. No special software is needed for the acquisitions. The initial velocity encoding (VENC) level was estimated based on echocardiographic information and was subsequently optimized, either in the presence of aliasing or when the difference between the initially set VENC and the determined maximal velocity was > 25%^8^. In the aortic and pulmonary position, the VENC was optimized during systole, whereas in the tricuspid position during diastole. Aliasing occurs in tricuspid valve during systole due to higher regurgitant velocity compared with inflow velocity. The potential for background phase offset errors was reduced by ensuring that the region of interest (ROI) in the imaging slice was for all PC sequences aligned in the isocenter of the magnet to minimize magnetic field inhomogeneities^5^. In all flow quantification, effective compensation for background phase offset was applied using adaptive image filtering. The background phase offset error after compensation was below the current limit of acceptance, i e < 0.6 cm/s, in all PC images of the present study^9^.

### CMR image analysis

Image analysis was performed using ViewForum (Philips Healthcare, Best, The Netherlands) and guided by the current international recommendations^10^. All analyses are performed using software for clinical routine. Aortic and pulmonary flow was determined by delineating the ROI on the respective magnitude image, copied onto the phase image and propagated through all phases using a semi-automated tracing algorithm, followed by manual adjustment if necessary. Delineation of the tricuspid valve was performed manually for all phases and in case of a closed valve, flow was zeroed by the delineation of an extremely small ROI on the closed valve. By integrating the velocity of each pixel in the delineated ROI over one heart cycle, the respective flow information was derived^11^.

LV volumes were obtained by manual tracing of the endocardial contour in end-diastole in the successive SA slices of the continuous SA stack, propagated through all phases using a semi-automated tracing algorithm, followed by manual adjustment, if necessary. Basal through-plane motion was compensated for according to a previously described technique where slices are considered to be within the LV when the blood volume is surrounded by 50% or more of ventricular myocardium^10^. RV volumes were acquired by manual tracing of the endocardial contour in the end-diastolic and end-systolic frames in the successive SA slices of the continuous SA stack. In the basal slice, only portions below the pulmonary valve were included in the volume.^10^. Papillary muscles and trabeculae were included in both the LV and RV cavity of each slice^10^. The end-diastolic (EDV) and end-systolic volume (ESV) was automatically computed by the slice summation technique for both ventricles. The LVSV, RVSV and ejection fraction (EF) were calculated as SV = EDV-ESV, and EF = SV/EDV x 100%, respectively.

### Reproducibility

Inter-observer variability of phase contrast flow measurement with BH and FB approach was assessed by two independent observers (S. A. G and C. L. P) in 10 healthy subjects and 10 patients with TR. The observers were blinded to previous results.

### Echocardiography

Two-dimensional echocardiography was performed using an Vivid E9 or E95 imaging system (GE Healthcare, Milwaukee, Wisconsin, USA). Image analysis was performed using EchoPAC (GE Healthcare, Milwaukee, Wisconsin, USA). Image acquisition and analysis was performed according to current echocardiography guidelines using a multi-parametric integrative approach^3^. The echocardiographic criteria to discriminate severe from moderate TR are PISA radius throughout the systole > 0.9 cm at Nyquist limits 30–40 cm/s, systolic reversal of hepatic vein flow, vena contracta width ≥ 0.7 cm, EROA > 0.4 cm^2^ RVol ≥ 45 ml, dilated RV with preserved function, dense and triangular continuous Doppler jet, large central jet > 50% of right atrium and dilated annulus with no leaflet coaptation or flail leaflet.

### Statistics

Values are expressed as the mean ± standard deviation (SD), unless otherwise stated. Correlation was assessed using the Spearman`s rank correlation coefficient (r). The intra-valvular differences between each breathing protocol was assessed using the Wilcoxon signed rank test. P-values < 0.05 were considered as statistically significant. Agreement between the BH and FB approach was described using Bland-Altman plots by calculating the mean difference ± SD and limits of agreement (LoA, mean difference ± 1.96 SD). Furthermore, the agreement between the BH and FB approach was assessed by intraclass correlation coefficient (ICC), calculated using a two-way mixed-effect model with an absolute agreement definition. Friedman´s ANOVA test was used for inter-valvular comparisons in each breathing state. In case when the null hypothesis was rejected (overall P value < 0.05), a post hoc analysis using the Wilcoxon signed rank test was applied. Using the Bonferroni correction for multiple testing, the null hypothesis was rejected if the P value was < 0.016. Inter-observer variability of flow volume measurements of CMR was assessed by an independent analysis of a second observer. The variability between two observers was described by the coefficient of variation ((SD of differences between the observer measurements/mean of observer measurements) x 100) and the repeatability coefficient [defined as 1.96 x √ (sum of the squares of the differences between observer measurements/n)]. The significance of the squared differences in the repeatability coefficient was assessed by the Wilcoxon signed rank test. Statistical analysis was performed using SPSS Version 27 (IBM, Armonk, NY, USA).

## Results

The characteristics of the entire study population are summarized in Table 1. The healthy subjects were much younger than the patients with TR (*P* < 0.001). All healthy subjects and patients with TR had normal LVEF except three patients (12%) with LVEF 35–44%. All healthy subjects and patients with TR had normal RVEF. Among the patients with TR, 11 (44%) had primary and 14 (56%) had secondary TR. Fourteen patients with TR (56%) were symptomatic and 10 (40%) had NYHA class > I. Twelve patients had severe TR (48%) and 13 patients (52%) moderate TR according to the integrative echocardiographic grading^3^.

**Table 1:** Characteristics of the study populations.

	Healthy subjects (*n* = 20)	Tricuspid regurgitation (*n* = 25)
Age (years)	31 ± 7	54 ± 14
Female, n (%)	9 (45%)	15 (60%)
Body surface area (m^2^)	1.80 ± 0.19	1.89 ± 0.22
Heart rate (beats/min)	63 ± 11	75 ± 13
SBP (mm Hg)	120 ± 7	135 ± 22
DBP (mm Hg)	75 ± 5	77 ± 11
Tricuspid annular dilation n (%)	-	8 (32%)
Tenting / tethering n (%)	-	6 (24%)
Tricuspid valve prolapse, n (%)	-	6 (26%)
Pulmonary hypertension	-	8 (32%)
History of heart transplantation	-	5 (20%)
History of endocarditis, n (%)	-	3 (12%)
History of chest radiation therapy	-	3 (12%)
Symptoms, n (%)	-	14 (56%)
Cardiovascular risk factors, n (%)		
Coronary artery disease	-	3 (12%)
Hypertension	-	7 (28%)
Hyperlipidemia	-	1 (4%)
Diabetes mellitus	-	1 (4%)
Enlarged right ventricle, n (%)	-	9 (36%)
CMR TR RVol ≥ 45 ml	-	9 (36%)

Tricuspid annular dilation defined as annular diameter > 21 mm/m^2^ in apical 4 chamber view. RVol, regurgitant volume = right ventricular stroke volume (RVSV) - pulmonary forward flow volume (PuFF).

**Fig. 2:**
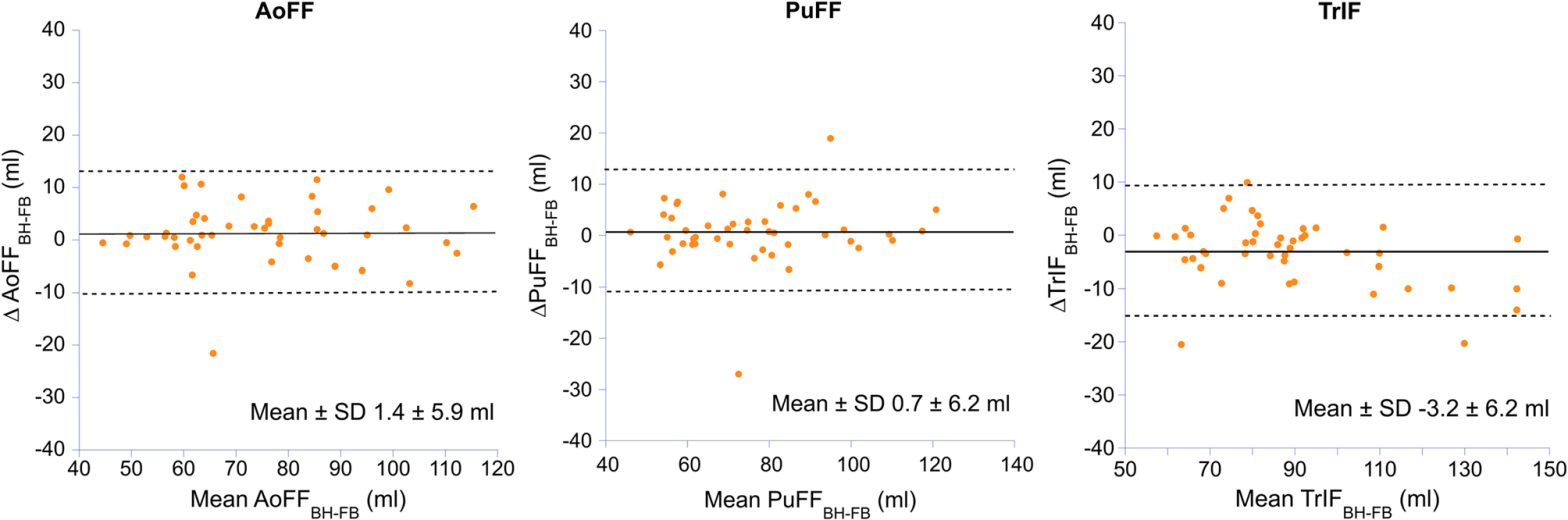
Bland Altman plots depicting the relation between flow volume measurements during free breathing and conventional breath-holding protocol in healthy subjects (*n* = 20) and patients with tricuspid regurgitation (*n* = 25).

**Fig. 3:**
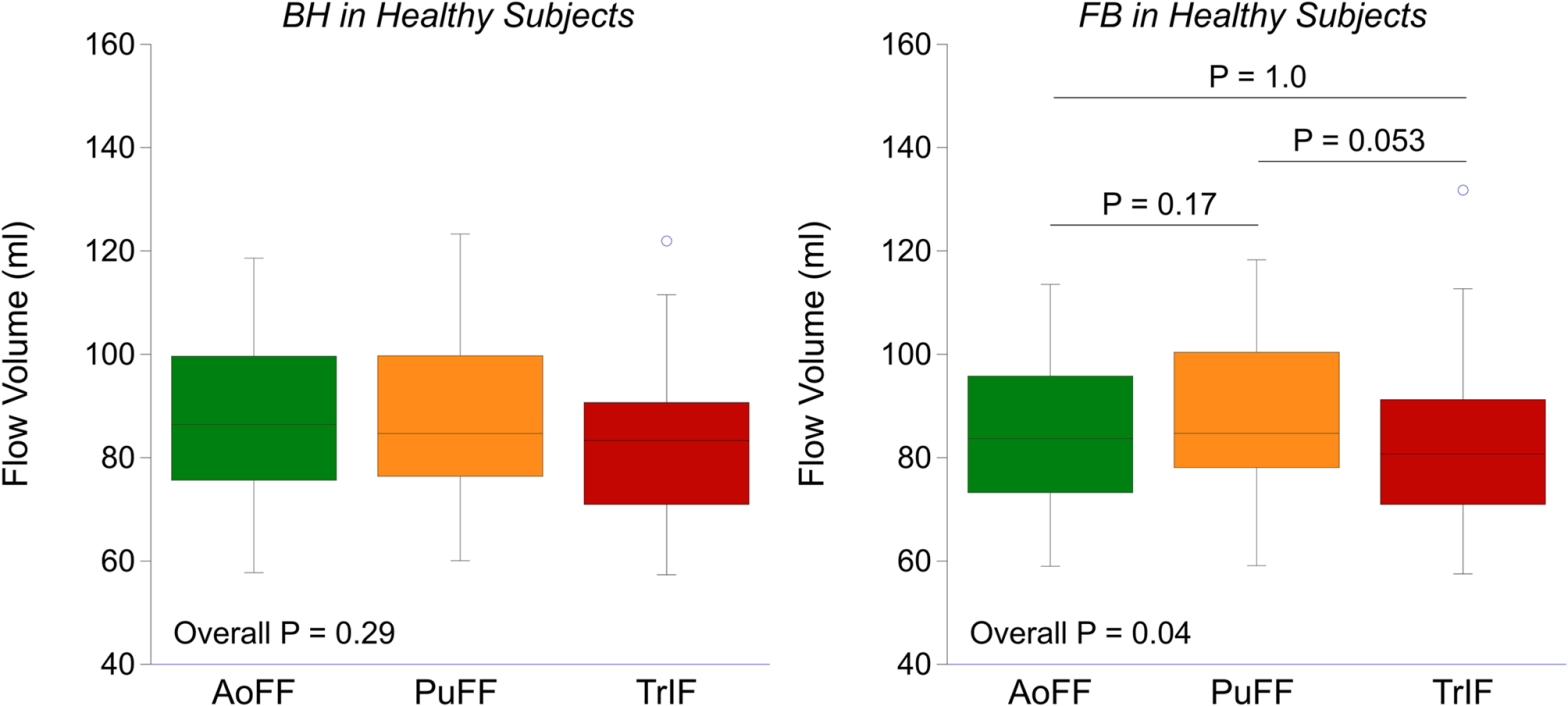
Boxplots comparing forward flow volumes in the aortic, pulmonary and tricuspid valve position during breath-holding and free breathing in healthy subjects (*n* = 20).

Flow volume acquisitions achieved good quality in all the study participants using both BH and FB approach (feasibility 100%). The overall coefficient of variation of the repeated flow measurements in the aortic, pulmonary and tricuspid position during BH was 5%, 4% and 7%, respectively. The correlation between flow volume measurements with FB and BH was strong to very strong, with r values > 0.9 with only two exceptions (Table 2). Mean difference ± SD between FB and BH were small and the ranges of the LoA were narrow (Table 2; Fig. 2). The ICC between the two breathing approaches were high, slightly higher in healthy subjects compared with patients (Table 3). The AoFF and PuFF were not significantly different between the BH and FB approach, whereas the TrIF was slightly higher during FB compared with BH in the entire study population (*P* < 0.01) and in patients with TR (*P* < 0.001, Table 2), but not in the healthy subjects. In the healthy subjects, inter-valvular comparisons demonstrated no significant differences during BH and FB (Fig. 3).

**Table 2:** Comparisons of flow volume (ml) measurements between BH and FB protocols in aortic, pulmonary and tricuspid valve position in healthy subjects (*n* = 20) and patients with tricuspid regurgitation (*n* = 25).

	BH	FB	MD ± SD	LoA	*r*	*P*
*AoFF*						
Total	76 ± 18	74 ± 18	1.4 ± 5.9	-10.1–13.0	0.92	0.11
HS	86 ± 17	85 ± 16	1.4 ± 4.1	-6.6–9.4	0.96	0.15
TR	67 ± 15	66 ± 15	1.5 ± 7.1	-12.4–15.4	0.80	0.30
*PuFF*						
Total	77 ± 19	76 ± 19	0.7 ± 6.2	-11.3–12.8	0.92	0.42
HS	88 ± 18	88 ± 17	0.41 ± 3.5	-6.4–7.3	0.96	0.60
TR	68 ± 15	66 ± 14	1.0 ± 7.7	-14.1–16.1	0.82	0.18
*TrIF*						
Total	88 ± 21	91 ± 24	-3.2 ± 6.2	-15.3–8.9	0.96	0.01
HS	84 ± 18	85 ± 19	-0.89 ± 4.1	-8.9–7.1	0.95	0.34
TR	90 ± 24	95 ± 26	-5.1 ± 7.0	-18.7–8.6	0.95	0.001

AoFF, aortic forward flow volume; BH, breath-holding; FB, free-breathing; HS, healthy subjects; LoA, limits of agreement; MD, mean difference of BH-FB; PuFF, pulmonary forward flow volume; SD, standard deviation; Total, the entire study population; TrIF, tricuspid inflow volume; TR, patients with tricuspid regurgitation.

**Table 3:** Intraclass correlation coefficient of flow volume measurements between BH and FB approach in aortic, pulmonary and tricuspid valve position in healthy subjects (*n* = 20) and patients with tricuspid regurgitation (*n* = 25).

	Intraclass Correlation (95% CI upper – lower bound)
*AoFF*	
Total	0.97 (0.95–0.99)
HS	0.98 (0.96–0.99)
TR	0.94 (0.87–0.97)
*PuFF*	
Total	0.97 (0.95–0.99)
HS	0.99 (0.98–1.00)
TR	0.92 (0.83–0.97)
*TrIF*	
Total	0.98 (0.94–0.99)
HS	0.99 (0.97–1.00)
TR	0.97 (0.88–0.99)

CI, confidence interval; otherwise as in Table 2.

The inter-observer variability of the flow volume measurements between two independent observers is summarized in Table 4. In general, the coefficient of variation and the repeatability coefficient were low and slightly lower in the semi-lunar valves in comparison with the tricuspid valve.

**Table 4:** Inter-observer variability of the flow quantification in aortic, pulmonary and tricuspid valve positions in healthy subjects (*n* = 10) and patients with TR (*n* = 10).

	Inter-Observer Variability	
	CV	RC
AoFF_BH_	2	2
AoFF_FB_	3	5
PuFF_BH_	2	2
PuFF_FB_	3	4
TrIF_BH_	5	10
TrIF_FB_	4	8

Data are expressed in %. CV, coefficient of variation; RC, repeatability coefficient. Otherwise, abbreviations as in Table 2.

## Discussion

The present study demonstrated good agreement of PC measurements in the aortic, pulmonary and tricuspid valve position using the FB and BH approach. Our findings indicate that the two breathing approaches can be used interchangeably in the semilunar valves, whereas FB could be more physiologic in the tricuspid valve position due to a higher respiratory dependence, particularly in patients with TR.

Valvular regurgitation, particularly of mild or less grade, is a frequent echocardiographic finding that does not impact the long-term survival. In contrast, moderate or severe valvular regurgitation has been associated with worse outcome and increased morbidity and mortality^1,12^. In the current management guidelines of VHD, severity grading is a highlighted step crucial for patient selection to intervention and echocardiography is the recommended primary diagnostic modality applying a multi-parametric integrative approach^2,3^. As such, the multi-parametric echocardiographic approach has intrinsic drawbacks including few feasible parameters supporting the final integration, and conflicting results of the individual parameters preventing a conclusive severity grading^13^. In the current guidelines, CMR plays an increasingly important role in the diagnostic work-up of valvular regurgitation as a secondary modality when echocardiography is inconclusive^14,15^. CMR is a powerful tool for flow volume assessment with high feasibility, precision, and reproducibility in phantom studies and in vivo in optimal flow condition for quantification^16^. In the recent years the emergence of transcatheter treatment has significantly enlarged the patient population eligible for valve intervention by including patients unsuitable for surgery. Conceivably, this has expanded the patient population undergoing CMR, which now also includes elderly frail patients and patients with heart failure and multiple comorbidities. In this population, CMR with its long scan times and repeated BH can be challenging. Inability to cooperate during the examination significantly reduces the image quality leading to reduced precision and increased variability, which consequently could impede correct severity grading of valvular regurgitation. This is partly addressed by the recent fast-evolving development in CMR technology promoting and focusing on shorter scan time and patient comfort^17,18^.

In the present study, a retrospectively navigated PC sequence, the most applied clinical routine flow technique, was evaluated during FB in the context of valvular flow assessment. Importantly, the present findings indicate that FB flow volume quantification has an excellent feasibility in the tricuspid as well as the semilunar valves and there were good agreements with the conventional expiratory BH approach. Furthermore, we demonstrated that the biases of the flow volumes acquired during FB and BH in the semilunar valve positions were small in both healthy subjects and patients, and that these two approaches can be used interchangeably. This finding corroborates with a few previous studies in healthy subjects showing good agreement between gentle expiratory BH and FB approaches^19,20^. However, the studies including patients with VHD are scarce. Good agreement was shown in a study in patients with left heart valve diseases^4^, whereas other studies have shown contradictory results. Johansson et al. investigated adult patients with a previous repaired tetralogy of Fallot and residual pulmonary regurgitation^21^. They found larger pulmonary forward flow during expiratory BH compared with FB. In contrast, Bolen et al. demonstrated that aortic volume flow was larger during FB compared with expiratory BH in patients with a clinical indication for CMR of thoracic aorta^22^. Thus, in patient populations the results of comparisons between FB and BH approach are divergent, and the underlying mechanisms have not been clearly elucidated. Interestingly, despite slightly less distinct flow images during FB compared with BH, the inter-observer variability of FB flow assessment is low and comparable to the conventional expiratory BH approach in the present study. This is crucial for facilitating the implementation of the FB approach in the clinical setting of the VHD.

In our study, there was a small but significantly lower TrIF during BH compared with FB in the patients with TR, but not in the healthy subjects, suggesting a higher respiratory dependence of the tricuspid inflow in the TR patients. Indeed, this is in line with our experience from echocardiography. A previous echocardiographic study has investigated the mechanism behind the dynamic nature of TR and its respiratory changes^23^. Topilsky and colleagues found in patients with mild or larger TR that the inspiratory increase of tricuspid regurgitant volume correlates with the geometric changes of the tricuspid annulus, sub-annular apparatus and RV. It is conceivable that the expiratory BH in the conventional CMR protocol abolishes the inspiratory changes in patients with TR, which may explain the slightly lower TrIF during expiratory BH compared with FB in the present study. This finding suggests that FB is the more physiologic approach and could be advantageous in patients with TR. However, the significance of FB to prevent underestimation of TrIF and its impact on the TR severity grading needs to be further investigated and confirmed in a larger patient cohort.

There are several limitations in the present study. Firstly, this is a single center study with a relatively small study cohort, which nevertheless was sufficient for our methodological comparisons and is in line with previous studies. It is certainly of interest to study the FB approach in other patient groups such as aortic and mitral regurgitation, but the present study population is included in a prospective multi-modality diagnostic study of TR that is still ongoing. Consequently, recruiting patients with other valve lesions was not possible in conjunction with the present study design. Secondly, given that our primary focus was to evaluate the methodological differences of FB and BH approach in the setting of valvular regurgitation, we decided to minimize other sources of variability such as arrythmias and included solely study subjects with regular heart rhythm. However, atrial fibrillation is common and frequently present in patients with mitral and tricuspid regurgitation. The usefulness of CMR FB approach in patients with atrial fibrillation needs to be evaluated in future patient-focused studies. Lastly, the present investigation examined the conventional 2D PC flow technique that is used in most clinical routine examinations. Comparisons with the real time and 4D PC flow techniques were not performed, but a recent study has demonstrated good agreement between these techniques^24^.

In conclusion, the present study demonstrated good agreement of flow volume measurements in the aortic, pulmonary and tricuspid valve position using FB compared with a conventional BH approach. Our data indicate that the two approaches can be used interchangeably in the semilunar valves, whereas FB could be more physiologic in the tricuspid valve position due to a higher respiratory dependence. However, the clinical significance of FB regarding the severity grading of TR needs to be confirmed in future studies. Thus, the FB approach of flow quantification can be an important diagnostic alternative that improves feasibility without sacrificing precision. It could be particularly useful in all patients with difficulties to hold their breath.

## Data Availability

The datasets generated during and/or analysed during the current study are available from the corresponding author on reasonable request.
